# Dialyzing a Brain-Dead Individual for Organ Procurement

**DOI:** 10.7759/cureus.56960

**Published:** 2024-03-26

**Authors:** Ripudaman S Munjal, Jaskaran Munjal, Gagandeep Dhillon, Venkata S Buddhavarapu, Harpreet Grewal, Pranjal Sharma, Ram K Verma, Ruth Lee, Rahul Kashyap

**Affiliations:** 1 Nephrology, Kaiser Permanente, Stockton, USA; 2 Internal Medicine, Shri Ram Murti Smarak Institute of Medical Sciences, Bareilly, IND; 3 Internal Medicine, University of Maryland Medical Center, Glen Burnie, USA; 4 Hospital Medicine, Banner Health, Phoenix, USA; 5 Radiology, Florida State University College of Medicine, Pensacola, USA; 6 Clinical Research, Global Remote Research Scholars Program, St Paul, USA; 7 Nephrology, Premier Renal Care Associates, Cuyahoga Falls, USA; 8 Internal Medicine/Nephrology, Northeast Ohio Medical University, Rootstown, USA; 9 Sleep Medicine, Parkview Health System, Fort Wayne, USA; 10 Nephrology, University of California, Davis, Davis, USA; 11 Research, Global Remote Research Scholars Program, St Paul, USA; 12 Research, WellSpan Health, York, USA

**Keywords:** dialysis, organ transplantation, extracorporeal kidney-replacement therapy, organ procurement, dialyzing brain-dead patients

## Abstract

Many patients are unable to receive organ transplantation as there is an expanding gap between the number of patients waiting for an organ and the number who receive it. Organ procurement from the brain-dead can address this expanding gap, especially because one brain-dead patient can potentially donate multiple organs to several recipients. Here, we describe a rare case of a previously healthy 26-year-old male who was declared brain dead after a motor vehicle accident but underwent hemodialysis to treat his acute kidney injury and hyperkalemia before successfully donating his heart and left kidney.

## Introduction

Organ transplantation has been increasing worldwide, although the availability of organs for transplantation remains limited [1,2]. Over the past few decades, the number of patients added to the organ donor waitlist has increased substantially, with reports as high as 154% between 2001 and 2021 [3]. Some also estimate that for every 10 minutes that pass, one patient is added to the transplant list, and 20 patients die per day waiting for a transplant [4]. One strategy for addressing this disparity between the apparent need for transplants and the growing waitlist is to retrieve organs from the brain-dead [5]. In contrast to living donors who can donate organs like the liver or kidney, a special benefit of organ procurement from the brain dead is that they can donate their heart or lungs, organs that could only be obtained if they are already dead [6]. The appropriate medical management of brain-dead organ donors is crucial for successful organ survival after donation because all potential organs must be functioning in their normal physiological condition prior to the time of retrieval [7,8].

Here we present the case of a 26-year-old male who was admitted after a motor vehicle accident and underwent multiple interventions but was eventually declared brain dead. Immediately after he was declared brain dead, he received kidney replacement therapy and was able to successfully donate his organs. He self-identified as an organ donor prior to his brain death, and his family consented to kidney replacement therapy prior to transplantation.

## Case presentation

A previously healthy 26-year-old male was admitted to the hospital after a high-velocity motor vehicle accident in which he was ejected from his vehicle and run over by another vehicle. He suffered multiple injuries, including extensive intracranial hemorrhage with transtentorial herniations, liver and right kidney injury, a C7 right transverse process fracture involving transverse foramen, an open book pelvic fracture, bilateral paired rib fractures, bilateral pneumothoraxes, pulmonary contusions, and complex extensive craniofacial fractures above his left calvarium and right temporal bone.

**Figure 1:**
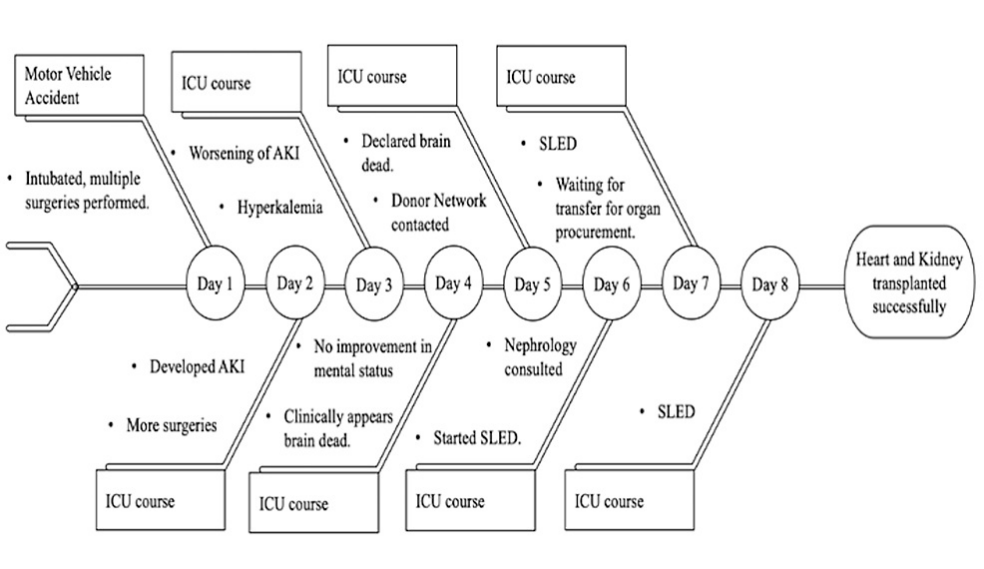
Hospital course This represents the ICU course for the patient. The patient was started on extracorporeal kidney replacement therapy in the form of SLED. CKRT was not available at our center. The patient was eventually transferred to a center capable of delivering a higher level of care, where his organs (heart and left kidney) were procured and successfully transplanted. AKI, acute kidney injury; CKRT, continuous kidney replacement therapy; SLED, sustained low-efficiency dialysis

His hospital time course is summarized in Figure 1.

On admission, he had a Glasgow Coma Scale of 3 and was intubated on arrival. That same day (Day 1), he underwent exploratory laparotomy, hemostasis of liver injuries, abdominal packing, repair of left eyebrow laceration, hemostasis of left frontoparietal scalp laceration, and repair of left frontoparietal scalp laceration. On Day 2, he underwent craniectomy for subdural hematoma evacuation, re-exploratory laparotomy, and hepatic angiography with embolization. He subsequently developed acute kidney injury (AKI) with hyperkalemia. His hospital stay was complicated by severe hypotension, which required multiple vasopressors, including the simultaneous administration of epinephrine, vasopressin, norepinephrine, and phenylephrine. On Day 4, there was no improvement in his mental status, and he was declared brain dead on Day 5 after a nuclear medicine cerebral perfusion scan, which showed absent supratentorial cerebral blood flow. The donor network was contacted for organ procurement, and he was started on IV levothyroxine while continuing his ventilator support. His AKI continued to worsen, with his creatinine peaking at 10.1 mg/dL (normally 0.5-1.4 mg/dL), likely as a result of acute tubular necrosis as noted on urine sediment with muddy brown casts. He subsequently became volume overloaded and hyperkalemic, with a serum potassium level of 7.0 mmol (3.5-5.1 mmol). Nonetheless, the patient was still deemed a suitable candidate for heart and left kidney donation, and nephrology was consulted for physiological stabilization before organs could be procured.

## Discussion

Organ transplantation is a surgical procedure in which the recipient’s nonfunctional organ is replaced with a donor’s healthy organ and can significantly enhance the recipient’s quality of life and life expectancy. The kidney is the most transplanted solid organ, followed by the liver and heart [4,9]. In 2021, despite the total number of candidates on the kidney transplant list surpassing 139,000 [10], only about 18% of kidney transplants took place, and of those, about 78% of those transplants were from deceased donors [10]. Although a large disparity exists between supply and demand for kidney transplantation, the number of deceased donor kidneys recovered for transplant but discarded is still high [11]. Nearly half of all discarded kidneys from 2010 to 2020 were from deceased donors with AKI, which frequently occurred because of hemodynamic and electrolyte instability [12].

To address the organ donation pool shortage, strategies have been developed to optimize the procurement of organs from brain-dead patients, like extracorporeal life support [13,14]. Specifically with the kidneys, however, kidney replacement therapy is rarely started after the diagnosis of brain death to treat the donor’s AKI [15]. Although not well studied, two kidney replacement therapies are currently present for kidney procurement from the brain-dead: continuous kidney replacement therapy (CKRT) and extracorporeal kidney replacement therapy [15].

In this presented case, our brain-dead patient received extracorporeal kidney replacement therapy in the form of sustained low-efficiency dialysis because CKRT was not available. The blood flow rate was kept at 200 ml/min to minimize the risk of hemodynamic instability, and his hyperkalemia and volume overload eventually resolved. The patient underwent hemodialysis for three consecutive days, enabling the successful procurement of his heart and kidney [16].

## Conclusions

In this case report, we aim to highlight the need for further investigation into kidney replacement therapy after death as a means of stabilizing patients for organ procurement, with the ultimate goal of addressing the organ shortage. Any intervention or treatment provided after death also creates an ethical dilemma regarding whether that treatment is in line with the patient’s wishes prior to brain death. Future studies should focus on developing guidelines around the use of extracorporeal kidney replacement therapy in brain-dead patients for organ procurement.
